# Intra-Articular Osteoid Osteoma Mimicking Juvenile Arthritis

**DOI:** 10.1155/2014/912609

**Published:** 2014-07-17

**Authors:** Sidi Yaya Traore, Dana Ioana Dumitriu, Pierre-Louis Docquier

**Affiliations:** ^1^Computer Assisted Robotic Surgery (CARS), Institut de Recherche Expérimentale et Clinique (IREC), Université Catholique de Louvain, Tour Pasteur +4, Avenue Mounier 53, 1200 Brussels, Belgium; ^2^Service de Chirurgie Orthopédique et Traumatologique, Cliniques Universitaires Saint-Luc, Avenue Hippocrate 10, 1200 Brussels, Belgium; ^3^Service de Radiologie et d'Imagerie Médicale, Cliniques Universitaires Saint-Luc, Avenue Hippocrate 10, 1200 Brussels, Belgium

## Abstract

In case of intra-articular osteoid osteoma, misdiagnosis as juvenile arthritis may occur, delaying adequate treatment. We report cases of intra-articular osteoid osteomas in children that were misdiagnosed and initially inappropriately treated with intra-articular corticoid injection. Diagnosis of osteoid osteoma was finally given by CT-scan and appropriate treatment by radiofrequency ablation or surgical ablation was performed. Clinicians and radiologists should be aware of the potentially confusing clinical and imaging findings associated with intra-articular osteoid osteoma.

## 1. Introduction

Osteoid osteoma (OO) is a small benign tumor observed in children and young adult patients described for the first time by Berstrand in 1950 and defined by Jaffe in 1953 [1]. It represents 10% [2] of all benign skeletal tumors and is preferentially found in the diaphysis cortex of the femur, followed in frequency by the tibia [3]. Intra-articular osteoid osteoma is uncommon, accounting for approximately 12% of all osteoid osteomas, and the most common site is the hip [4].

The typical clinical manifestations are nocturnal pain, unrelated to physical activity, increased by rest, and relieved by aspirin. The tumor is composed of a nidus of vascular osteoid tissue and woven bone lined by osteoblasts. It is frequently associated with considerable surrounding inflammation. The diagnosis is usually straightforward when imaging reveals a radiolucent nidus surrounded by variable degrees of reactive sclerosis. However, the diagnosis can be elusive when osteoid osteoma occurs in atypical locations, as they may have a nonspecific and misleading appearance on different imaging modalities, particularly on MRI [5]. Atypical juxta-articular or intra-articular location may mimic the clinical manifestations of arthritis or inflammatory synovitis, leading to misdiagnosis. The reported delay of diagnosis is variable in the literature [6–8]. The diagnosis is achieved by a combination of clinical data, radiograph, CT-scan, MRI, and bone scan. In young patients, the removal of these intra- or juxta-articular lesions is necessary to prevent complications as growth disturbance or joint incongruency.

CT-guided percutaneous ablation is now widely performed (radiofrequency ablation or laser ablation).

We report cases of intra-articular OO initially diagnosed as juvenile arthritis in which misdiagnosis led to inappropriate treatment (Table 1).

## 2. Case Presentation

*Case 1.* This 14-year-old girl initially suffered from type A streptococcal angina, without temperature. Two weeks later, she complained from a right hip pain, which was improved but not relieved by nonsteroidal anti-inflammatory drugs (NSAIDs) (naproxen 250 mg 2 times a day). The pain was present at night and at rest. She presented in another institution where a hip radiograph was found normal and a hip joint echography revealed aspecific hip joint effusion. The diagnosis of transient synovitis was given and treatment consisted of relative rest and NSAIDs. As the pain was persisting, blood serologies were analyzed and showed positive antibodies for* Mycoplasma pneumonia*. A treatment with clarithromycin was started.

**Figure 1:**
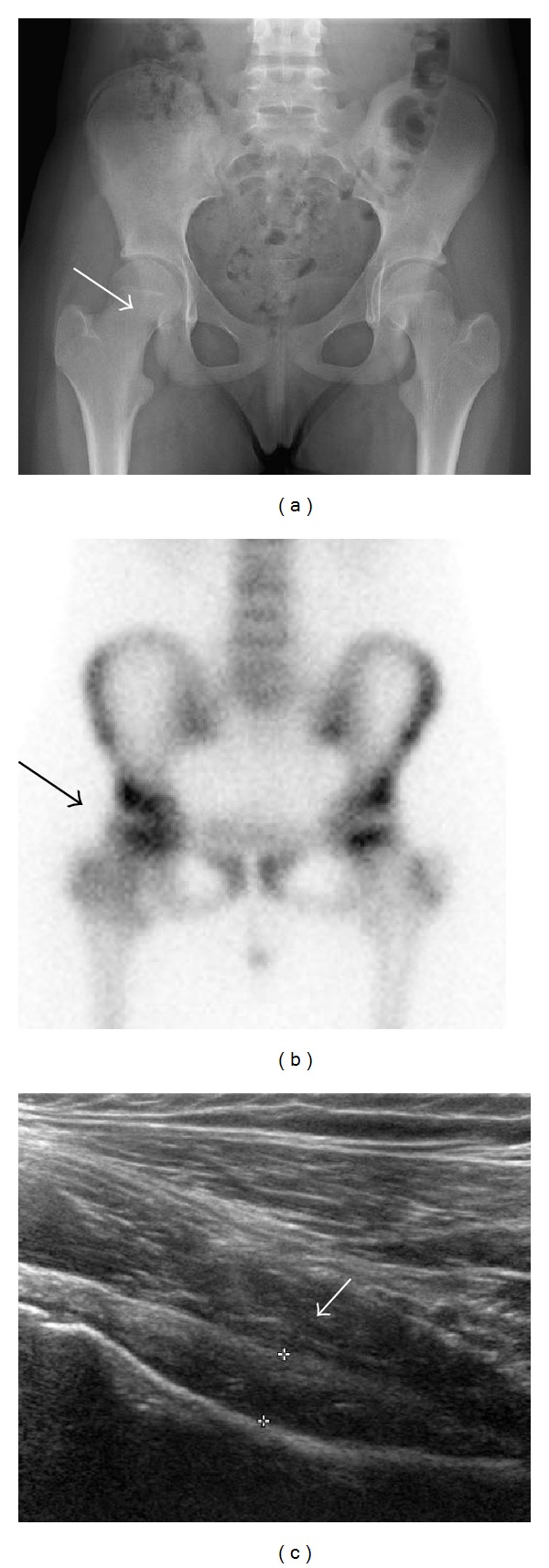
14-year-old girl with intra-articular osteoid osteoma of the right femoral neck. (a) Frontal X-ray of the pelvis, interpreted as normal. (b) Bone scan, demonstrating increased uptake of the right hip joint. (c) Hip ultrasound, longitudinal scan, showing synovial thickening of the anterior articular recess, right hip.

**Figure 2:**
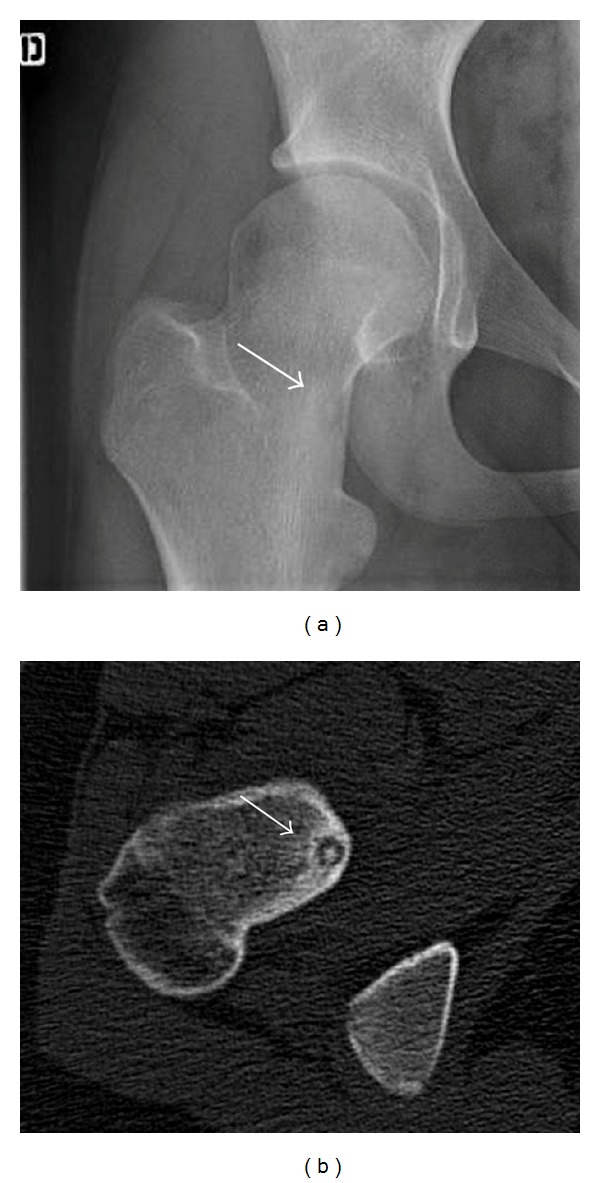
Same patient as in Figure 1. (a) A repeated frontal X-ray of the right hip, performed six months later, demonstrates a radiolucent nidus of femoral neck. (b) Confirmation was obtained by CT-scan.

Because of pain persistence 6 weeks later, she consulted our clinic for a second opinion. She had a painful range of motion limitation of the right hip. Hip radiography, hip echography, and bone scan were performed (Figure 1).* Borrelia, Mycoplasma, *and* Chlamydia* serologies were negative, as well as Rose-Waaler test (passive hemagglutination test for rheumatoid factor in the serum), latex agglutination test for rheumatoid factor, and HLA B27 test. Bone scan showed joint hyperfixation. The rheumatologist retained the diagnosis of monoarticular juvenile arthritis. A treatment by intra-articular methylprednisolone acetate injection under general anesthesia was performed but without symptoms improvement. One week later, she was reviewed with persistence of pain. A CT-scan and a new radiograph were performed (Figure 2). These exams confirmed OO diagnosis with a nidus of 5.5 mm in the femoral neck. The diagnosis was made with a delay of six months from the first consultation. A radiofrequency ablation was performed with good resolution of pain and recovery of a normal hip range of motion.

*Case 2.* The second patient was a 15-year-old girl first followed in another hospital. She presented with permanent right hip pain with night worsening and limping. These symptoms occurred without context of trauma, fever, or infection. A MRI showed joint effusion. No biological analysis (rheumatoid factor, HLA B27, and viral serologies) was made but the diagnosis of juvenile arthritis was retained. Treatment initially consisted of oral NSAIDs (diclofenac 50 mg 3 times a day), and later intra-articular methylprednisolone acetate injection under general anesthesia was performed without improvement of pain.

One month later, she was reviewed by the pediatric orthopedist with the same symptomatology. She was taking 4 g of paracetamol and 150 mg of diclofenac a day without improvement of symptoms. Clinical examination revealed decreased hip internal rotation and abduction. A bone scan was performed, showing right femoral neck hyperfixation. No argument was found in favor of an OO or osteomyelitis but a CT-scan was performed in the hyperfixation area. The CT-scan found a 6 mm nidus in the anterior edge of the femoral neck and surrounding hyperostosis. The diagnosis was made seven months after the onset of symptoms.

The patient underwent a radiofrequency ablation under general anesthesia. One-day hospitalization was necessary. The pain disappeared completely some day after the surgery. One month after surgery, the mobility of the hip was improved. Abduction and internal rotation became identical to the contralateral side. Only external rotation was less than the contralateral side (30°/45°). At the last followup the patient kept a slight decrease of the external rotation compared to the contralateral side. No recurrence of OO was observed after 2 years of followup.

**Figure 3:**
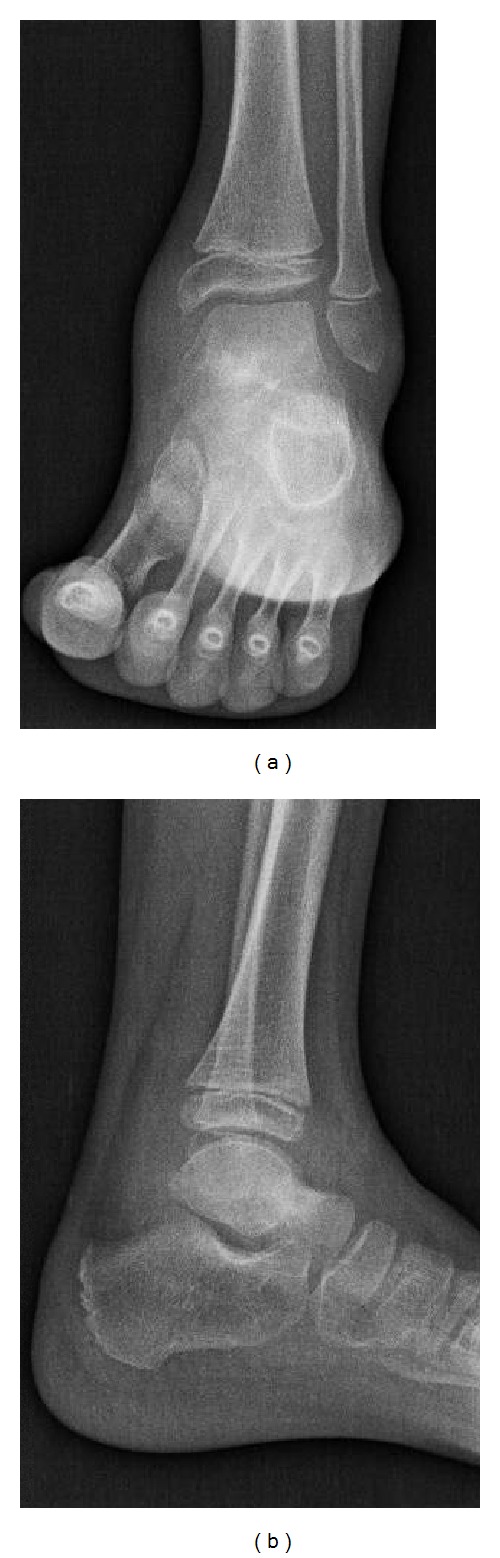
8-year-old boy with osteoid osteoma of the left talar neck. Synovial thickening of the ankle and subtalar joint was diagnosed with echography. X-ray showed ankle effusion but no bone anomaly was initially reported. However retrospective X-ray analysis showed a talar neck hyperostosis.

*Case 3.* The third patient was 8-year-old boy who presented in the emergency room of our hospital for left ankle pain. Pain was permanent with ankle swelling and limping but without fever. The mobility of the ankle was painful at clinical examination. Radiography was performed and was found normal (Figure 3). Echography revealed a joint effusion, synovial thickening, and reactive hyperemia of surrounding soft tissue. Diagnosis of inflammatory arthritis was retained but no blood biology was performed. He was first treated with oral NSAIDs (ibuprofen 3 times a day) and nonweight-bearing with crutches.

Four days later the patient was seen in general pediatric clinic with relative pain improvement but with persistence of skin redness and ankle swelling. The blood biology and urinary sediments did not found any argument of inflammatory pathology.* Borrelia*,* Cytomegalovirus*, Epstein-Barr virus, and parvovirusB19 serologies were performed. Only EBV IgG and parvovirusB19 IgG index were found positive. The diagnosis of juvenile arthritis was retained and the treatment with oral ibuprofen was continued.

He was seen 10 days later by pediatric rheumatologist without significant symptoms improvement. He also concluded to idiopathic juvenile arthritis. The patient underwent an intra-articular methylprednisolone acetate injection under general anesthesia and two weeks later additional methotrexate was given 10 mg a week with folic acid. No symptomatology improvement occurred.

**Figure 4:**
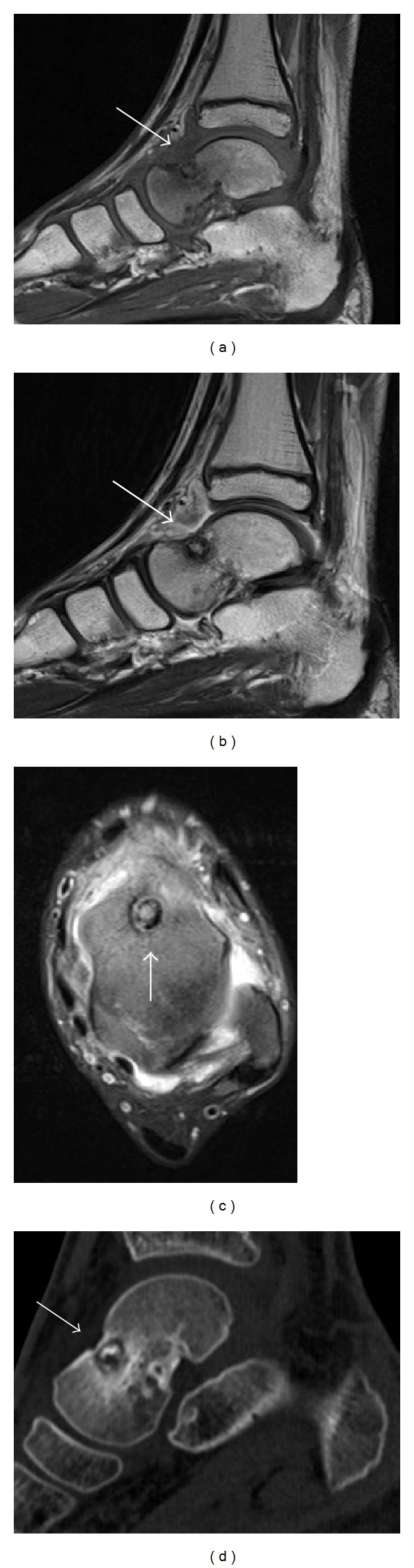
Same patient as in Figure 3. (a–c) MRI of the left ankle demonstrates the talar neck nidus, surrounded by abundant oedema and synovial inflammation ((a) sagittal T1-WI, (b) sagittal T2-WI, and (c) axial fat saturated PD-WI). (d) CT-scan of the left ankle also demonstrates the talar neck nidus.

Considering the persistence of symptomatology, a CT-scan and a MRI (Figure 4) were performed and gave the diagnosis of talar neck OO four months after the onset of pain. The nidus size was 8 mm in the dorsal aspect of the talar neck (Figure 4).

The patient had sustained a fracture of both forearm bones treated by elastic stable intramedullary nailing 6 months before. As the surgery of nails removal was already planned, he was operated at the same time by direct open curettage of the OO, under general anesthesia and in one-day surgery. Histopathology confirmed the diagnosis of OO. There was no postoperative complication and the pain disappeared quickly two days after surgery. One month after surgery the mobility of ankle was similar to the contralateral side and limping had disappeared.

*Case 4.* A 6-year-old girl was referred by her family doctor in rheumatology for right hip pain with limping. Pain was diurnal and nocturnal. There was no fever, not context of infection or trauma. There was a history of painful swelling of metacarpophalangeal joints of thumb and index finger and of the proximal interphalangeal joint of index finger.

Clinical examination showed painful hip motion, limitation of hip abduction and medial rotation, and also swelling of the homolateral knee. The knee was not painful.

C-reactive protein (CRP) was less than 0.6 mg/L (normal: <0.5 mg/L), protein electrophoresis and hemogram were normal. Antinuclear factor (ANF) test,* Borrelia* serology, and hip radiography were normal. Echography found joint effusion with synovial thickening and bone scan showed a diffuse hip hyperfixation. The retained diagnosis was juvenile arthritis and was initially treated by naproxen 250 mg a day.

Given the persistence of pain the patient was reviewed one month later by the rheumatologist and underwent an intra-articular methylprednisolone acetate injection with no change of symptomatology. Three weeks after the injection a new echography, radiography, and CT-scan were performed that confirmed the diagnosis of OO of femoral neck six months after the onset of pain.

After obtaining the consent of the parents, the patient underwent the radiofrequency ablation in one-day surgery.

Two days after the surgery the pain disappeared. One month later, hip joint range of motion was normal and limping had disappeared.

## 3. Discussion

Intra-articular OO are often misdiagnosed as juvenile arthritis and are initially inappropriately treated. Three cases of our intra-articular OO were localized in the hip, which is the more frequent location of intra-articular OO [4]. In our patients, the common clinical symptoms were pain, decrease of joint range of motion, limping, or soft tissue swelling [9]. These signs were sometimes improved but never relieved by NSAIDs.

Despite the improvement of the imaging techniques, diagnosis of atypical location of OO still remains a challenge. The initial erroneous diagnosis of juvenile arthritis was given in all of our cases and the delay before correct diagnosis ranged from 4 to 7 months after the onset of symptoms. In the literature, this delay is variable, ranging from 2 months to 10 years [6–8]. In a small series Szendroi et al. [10] found that for the intra- or juxta-articular location the duration of symptoms before the diagnosis (26.6 mo) was 3 times longer than for extra-articular sites.

In our cases, radiograph was not able to give the diagnosis of intra-articular OO and echography showed aspecific joint effusion. On initial radiograph the nidus is found in only 28 to 50% of the cases due to the minimal sclerotic reaction of the trabecular bone, especially when the size of the nidus is below 3 mm [6, 11].

Bone scan (scintigraphy) may orientate the diagnosis of OO. The distinctive presentation is commonly referred to as the “double density sign.” In the case of intra-articular OO, this “double density sign” is not often seen due to synovitis, osteoporosis, and hyperemia [6, 12]. In two of our cases, bone scan allowed the diagnosis, in association with CT-scan. In case of positive CT-scan, bone scan is not recommended any longer.

CT-scan is the method of choice to identify the nidus of an intra- or juxta-articular OO. The appearance on CT-scan is a round or oval low attenuation nidus surrounded by varying degrees of sclerosis. In all of our patients, this exam showed the nidus. Spouge and Thain showed that CT-scan may fail to diagnose OO when the nidus is in a cancellous bone location, due to the lack of perinidal bone sclerosis [13].

On MRI, the nidus is typically hypointense on T1- and T2-weighted images, with surrounding bone marrow edema and high contrast enhancement after gadolinium administration. Intra-articular lesions may demonstrate synovial thickening apparent on MRI, confirmed by contrast injection. However, precise location of the nidus may not be easy and in 35% of the cases, the nidus cannot be detected since it is often hidden by the associated perilesional edema surrounding the lesion, while in 50% of the cases, the nidus has an atypical presentation, which may lead to misdiagnosis [14]. Only in one of our cases, MRI was suggestive of the diagnosis of OO but CT-scan was necessary to confirm the diagnosis (Case 3).

All patients had a complete relief of pain after radiofrequency ablation or surgery and no recurrence was detected at the time of the last followup.

Clinicians and radiologists should be aware of the potentially confusing clinical and imaging findings associated with intra-articular osteoid osteoma.

## Figures and Tables

**Table 1:** Clinical data of the patients.

Sex	Age (years)	Initial symptoms	Location	Initial imaging	Initial treatment	Delay before correct diagnosis from symptoms onset
F	14	Permanent hip pain, limping, improved with NSAIDs, and decreased range of motion (ROM)	Femoral neck, intra-articular	(i) Radiograph: normal (ii) Ultrasound: aspecific joint effusion (iii) Bone scan: joint hyperfixation	(i) Naproxen 250 mg 2 times a day (ii) Corticoid intra-articular injection	6 months

F	15	Permanent hip pain, limping, night worsening, and decreased ROM	Femoral neck, intra-articular	(i) Radiograph: normal (ii) MRI: joint effusion	(i) Diclofenac 3 times a day (ii) Corticoid intra-articular injection	7 months

M	8	Permanent ankle pain, limping, night worsening, ankle swelling, skin redness, and decreased ROM	Talar neck, intra-articular	(i) Radiograph: ankle joint swelling (ii) Ultrasound: aspecific joint effusion	(i) Ibuprofen 3 times a day (ii) Corticoid intraarticular injection (iii) Methotrexate 10 mg a week (iv) Folic acid	4 months

F	6	Permanent hip pain, limping, and decreased ROM	Femoral neck, intra-articular	Radiograph: normal	(i) Naproxen 2 times a day (ii) Corticoid intra-articular injection	6 months
